# Integrating machine-learning and nanotechnology to quantify pH-modulated oxaliplatin release

**DOI:** 10.1038/s41598-025-26145-3

**Published:** 2025-11-26

**Authors:** Sonia Fathi-karkan, Abbas Rahdar, Maryam Shirzad

**Affiliations:** 1 Natural Products and Medicinal Plants Research Center, North Khorasan University of Medical Sciences, Bojnurd, 94531-55166 Iran; 2 Department of Advanced Sciences and Technologies in Medicine, School of Medicine, North Khorasan University of Medical Sciences, Bojnurd, 9414974877 Iran; 3 Food and Drug Research Center, Food and Drug Administration, Ministry of Health and Medical Education, Tehran, Iran; 4 Department of Physics, University of Zabol, Zabol, Iran; 5 Nanotechnology Research Center, Pharmaceutical Technology Institute, Mashhad University of Medical Sciences, Mashhad, Iran

**Keywords:** Oxaliplatin, Nanomicelles, Breast cancer, Drug delivery, Controlled release, pH-responsive, Machine learning, SHAP, Biochemistry, Biotechnology, Cancer, Chemistry, Drug discovery, Nanoscience and technology

## Abstract

The purpose of this work was to formulate and characterize pH-sensitive, surfactant-based nanomicelles for the targeted delivery of Oxaliplatin to breast cancer cells. A secondary aim was to utilize machine learning (ML) models to interpolate and dissect the sophisticated, pH-dependent drug release kinetics. Nanomicelles of Oxaliplatin were prepared by Pluronic F-127 through a thin-film hydration technique. The nanomicelles were characterized for size, morphology, encapsulation efficiency, and drug release profile in physiological (pH 7.4) and acidic, tumor-mimicking (pH 5.4) media. The MTT assay was used to test cytotoxicity against L929 normal fibroblasts and MCF-7 breast cancer cells. ML models (Random Forest, Gradient Boosting, SVR) were trained on experimental release data to anticipate crucial release phase changes using SHAP analysis. Prepared nanomicelles were monodisperse and spherical with hydrodynamic diameter 290.3 nm and encapsulation efficiency 40.2%. They had good pH-responsive release with cumulative release 77.5% and 43.5% at pH 5.4 and 7.4, respectively, in 96 h. Kinetic modeling revealed a shift from Fickian diffusion at pH 7.4 to anomalous transport at pH 5.4. ML models showed great interpolation performance (R² > 0.97), and SHAP analysis showed remarkable release transitions. The cytotoxicity assays were different from free Oxaliplatin with improved activity against MCF-7 cells and lower toxicity against L929 cells. Surfactant-based nanomicelles are an effective delivery platform for pH-directed delivery of Oxaliplatin to enhance its therapeutic index. Nanomedicine formulation design is enabled by ML through comprehensive release kinetics analysis.

## Introduction

Breast cancer remains a leading cause of cancer-related mortality in women worldwide, with a high annual incidence of new diagnoses. Although significant advancements have been made in treatment modalities, therapeutic efficacy continues to be hindered by drug resistance, poor selectivity, and severe side effects^1^. As a result, the therapeutic treatments become less effective, and this enhances combination therapy use in metastatic and triple-negative breast cancers. Resistance to drugs often develops overtime. Besides, the nonspecific nature of the traditional therapies often results in harmful side effects that constantly compromise the compliance of therapy and quality of life of patients. These challenges stress the urgent need for novel therapeutic strategies, including nanotechnology-based drug delivery systems (DDSs) and personalized medicine, to enhance the clinical outcomes for patients diagnosed with breast cancer^2,3^.

Oxaliplatin is a third-generation chemotherapeutic drug based on platinum with broad efficacy against a wide range of malignancies, including breast cancer. However, clinical use of this agent is usually restricted due to the common incidence of drug resistance and systemic toxicity^4^. Among various promising approaches, nanotechnology-based DDSs, especially surfactant-based nanomicelles, are gaining significant interest to elevate the stability, and targeted delivery of chemotherapeutic drugs including oxaliplatin. Such systems enhance the cellular intake as well as limit drug efflux, boosting the effectiveness and overcoming its resistance at a rather minimal systemic toxicity^5^. Nanomicellar incorporation of the drug allows for a wide therapeutic index with reduced adverse effects associated with oxaliplatin. The integration of nanotechnology within the delivery system will significantly increase both the efficacy and safety profiles of oxaliplatin in treating breast cancer conditions^6^.

Several studies in recent times have looked into the use of a range of nanocarrier systems to help the intracellular delivery of oxaliplatin. The micelles of chitosan/ vitamin E succinate loaded with oxaliplatin presented increased cytotoxic impact on MDA-MB-231 and MCF-7 human breast cancer cell lines, which is represented via the lower IC50 value against free oxaliplatin. These micelles also induced significant DNA damage, mitochondrial depolarization, apoptosis, and cell cycle arrest in the G2/M phase. In vivo examination further demonstrated the potential of these micelles as a nanomedicine to surmount conventional oxaliplatin-mediated drug resistance and toxicity in breast cancer therapy, showing significant reduction in nephrotoxicity and significant retardation of tumor growth, in comparison with free oxaliplatin^7^.

The present study aims to formulate and characterize oxaliplatin-loaded surfactant-based nanomicelles and evaluate their cytotoxicity on MCF-7 breast cancer cells. Incorporation within nanomicelles is expected to increase bioavailability and stability, thereby enhancing the therapeutic potential of oxaliplatin and providing a reliable and targeted DDSs. MCF-7 cells were chosen as a representative model of estrogen receptor-positive breast cancer, enabling assessment of selective anticancer efficacy while minimizing toxicity to normal fibroblasts. While traditional kinetic models (e.g., Higuchi, Korsmeyer-Peppas) are widely used, they often struggle to accurately capture the complex, multi-phasic release profiles typical of stimuli-responsive nanocarriers. These models rely on pre-defined equations that lack the flexibility to represent nonlinear dynamics effectively, identify critical release phase transitions, or integrate multiple influencing factors simultaneously. In order to go beyond these limitations, machine-learning (ML) techniques can serve as flexible, data-driven interpolators of release kinetics. While ML models can reconstruct observed experimental profiles with high apparent accuracy, they are limited by dataset size and prone to overfitting; thus, their outputs should be considered complementary to mechanistic kinetic modeling rather than predictive replacements. In this study, we adopt a dual strategy: first, we prepare and characterize oxaliplatin-loaded surfactant-based nanomicelles and evaluate their cytotoxicity. Then, we integrate experimental release profiles into ML models to generate smooth interpolations of pH-dependent release kinetics and highlight transition phases in the release curves. This combined experimental–computational framework demonstrates the value of ML as a descriptive and exploratory tool for analyzing drug release profiles. Through the combination of computational intelligence and experimental pharmaceutics, the current work seeks not only to elucidate the mechanisms underlying controlled oxaliplatin delivery but to present a rational design for formulating more effective and intelligent nanomedicine platforms for breast cancer therapy.

## Experimental sections

### Materials

In this study, chemical reagents and materials were analytical grade and were not further purified to maintain the experimental results consistent and reproducible. Sodium chloride, potassium dihydrogen phosphate, disodium phosphate dihydrate, and ethylenediaminetetraacetic acid (EDTA) were supplied by Sigma-Aldrich and used for buffer solutions to maintain pH and ionic strength. Oxaliplatin (≥ 98% purity, Sigma-Aldrich) was used as the drug, with Pluronic^®^ F-127 as the surfactant and ethyl butyrate as the oil phase (both from Sigma-Aldrich). Cell culture experiments were done with RPMI 1640 medium containing fetal bovine serum, penicillin-streptomycin, and trypsin-EDTA. MCF-7 and L929 murine fibroblast cells were obtained from the Pasteur Institute of Iran. MCF-7 was obtained from breast epithelial cells of a 69-year-old white woman with metastatic cancer. Fibroblasts from mouse connective tissue form the L929 cell line. Standards were used to cultivate all cell lines. The ethical norms of the source institution were followed for cell line purchase and use. The selectivity of the nanomicelles toward the cancer cells was investigated in comparison with L929 fibroblasts. All the experiments were conducted under sterile conditions to ensure reliability and reproducibility of the results.

### Methods

#### Preparation of oxaliplatin-loaded nanomicelles

Oxaliplatin-loaded nanomicelles were prepared by aqueous hydration and thin-film self-assembly process that was designed to accommodate oxaliplatin’s hydrophilicity. The process accommodates drug association with the amphiphilic polymeric system without the necessity of dissolution in a hydrophobic oil core. Pluronic F-127 and ethyl butyrate were co-dissolved in minimal volumes of ethanol using a molar ratio of 1:1.5. Ethyl butyrate here is not employed as a solvent for oxaliplatin but as a hydrophobic stabilizer, stabilizing the PPO blocks of Pluronic F-127 and aiding micelle formation. The solvent was evaporated under reduced pressure at 40 °C to give a thin surfactant/oil film, which was further dried under vacuum. Dry film was then hydrated with an aqueous solution of oxaliplatin in PBS (5 mg/mL, pH 7.4) and the concentration of Pluronic F-127 was finalized at 2.0% w/v. Aggressive stirring (800 rpm, 4 h) facilitated hydration and spontaneous self-assembly into nanomicelles with PPO/ethyl butyrate domains creating a hydrophobic core and PEO chains making up the corona. Because of hydrophilicity of oxaliplatin, it was entered largely by polar interactions and hydrogen bonding in the dense PEO corona rather than entrapping within the hydrophobic core. Finally, ultracentrifugation at 20,000 rpm for 60 min removed unassociated drug crystals, and the supernatant of purified oxaliplatin-loaded nanomicelles was collected for characterization.

#### Scanning electron microscopy (SEM) analysis

The topography and morphology of drug-free and Oxaliplatin-loaded nanomicelles were characterized by SEM. SEM was performed on TESCAN VEGA3 microscope with an accelerating voltage of 20 kV. The samples were cast by placing a diluted aqueous suspension of nanomicelles onto a silicon wafer and allowing to air-dry at room temperature. The dry samples were subsequently sputter-coated with a thin gold coating to enhance conductivity and to eliminate charging artifacts. Images were captured at a working distance of 5.74 mm and at 100,000× magnification, the optimal resolution for individual nanomicelles and size, shape, and distribution.

#### Particle size evaluation

Using Dynamic Light Scattering (DLS) and a Malvern Zetasizer Nano ZS at room temperature, the particle size and zeta potential of the oxaliplatin-loaded nanomicelles were determined. For these measurements, a solution of 1 mg/mL of the nanomicelles was dissolved in deionized water.

#### Efficiency of drug encapsulation

The encapsulation efficiency (EE%) and drug loading (DL%) of oxaliplatin-loaded nanomicelles were determined using UV–Vis spectroscopy (Shimadzu UV-1700 PharmaSpec, Kyoto, Japan). To separate free (unassociated) drug from micelle-associated drug, dispersions were centrifuged at 20,000 rpm for 60 min. The supernatant containing unassociated oxaliplatin was carefully collected, and its absorbance was measured at 255 nm, the characteristic absorption peak of oxaliplatin. Oxaliplatin concentration was quantified against a calibration curve prepared in PBS (pH 7.4). Encapsulation efficiency and drug loading were calculated according to:

1 $$\:\text{E}\text{n}\text{c}\text{a}\text{p}\text{s}\text{u}\text{l}\text{a}\text{t}\text{i}\text{o}\text{n}\:\text{e}\text{f}\text{f}\text{i}\text{c}\text{i}\text{e}\text{n}\text{c}\text{y}\:\left(\text{E}\text{E}\text{\%}\right)=\frac{({W}_{total}-\:{W}_{free})}{{W}_{total}}\:\times\:100\:\:\:\:\:\:\:\:\:\:$$

2 $$\:Drug\:Loading\:\left(DL\text{\%}\right)=\frac{({W}_{total}-\:{W}_{free})}{{W}_{nanomicelles}}\times\:100\:\:\:\:\:\:\:\:\:\:\:\:\:\:\:\:\:\:\:\:\:\:\:\:\:\:\:\:\:$$

where $\:{W}_{total}$ is the initial oxaliplatin content, $\:{W}_{free}$ is the amount of unassociated drug, and $\:{W}_{nanomicelles}\:$is the mass of lyophilized nanomicelles obtained from a known volume of dispersion.

#### Drug release study in vitro

The in vitro release of oxaliplatin from nanomicelles was compared according to the dialysis technique under physiological conditions. A milliliter of oxaliplatin-loaded nanomicelles was placed in a dialysis membrane (MWCO 12,000–14,000 Da) and suspended into 50 mL of phosphate-buffered saline (PBS) at pH 7.4 or 5.4. The aqueous solubility of oxaliplatin was determined to be sufficient for good quantitation under these conditions. The formulation was maintained at 37 °C under constant agitation at 90 rpm to simulate physiological conditions. Samples were removed at certain time intervals from the release medium and replaced with fresh PBS of the same pH. Oxaliplatin content was spectrophotometrically monitored at 255 nm on a calibration curve established in PBS. Cumulative percentage oxaliplatin released was calculated against total drug loaded into the nanomicelles.

#### Cell viability assay

MCF-7 breast carcinoma cancer cells and L929 normal fibroblasts were used to measure the cytotoxicity of free oxaliplatin and nanomicelle-carrying oxaliplatin. Cells were plated at 10⁴ cells/well in 96-well plates to provide for adhesion and preserved at 37 °C for 24 h in a humidified 5% CO₂ environment. Following incubation, cells were exposed to different concentrations of free oxaliplatin or nanomicelle-carrying oxaliplatin in five replicates (*n* = 5) to provide statistical consistency. The treated cells were further incubated at the same conditions for 48 h to measure cellular responses. Subsequent to treatment, 20 µL of 5-mg/mL MTT solution in PBS was added to every well, and cells were incubated at 37 °C for 4 h. Throughout this time, cells that were still alive reduced MTT into insoluble purple formazan crystals. The medium was aspirated, and 150 µL of DMSO was added to solubilize formazan crystals. Plates were shaken gently for 10 min to dissolve them thoroughly. Absorption was read at 570 nm using a BioTek microplate reader (Bad Friedrichshall, Germany). The percentage of cell viability was estimated based on the following formula:

3 $$\:Cell\:viability\:\left(\%\right)=\:\frac{\left(TAbsorbance\:of\:test\:sample\right)}{\left(Absorbance\:of\:control\:sample\right)}\times\:100\:\:\:\:\:\:\:\:\:\:\:\:\:\:\:\:\:\:\:\:\:\:\:\:\:\:\:\:\:\:\:\:\:\:\:\:\:\:\:\:\:\:\:\:\:\:\:\:\:$$

#### Machine learning (ML) studies

##### Data acquisition

Experimental oxaliplatin release data were collected under two pH conditions: acidic tumor-like (pH 5.4) and physiological (pH 7.4). Drug release was monitored at ten predefined time intervals (0, 1, 3, 6, 12, 24, 48, 72, and 96 h). The corresponding cumulative release percentages are presented in Table 1.

**Table 1:** Experimental oxaliplatin release profiles (% cumulative release) at pH 5.4 and pH 7.4.

Time (h)	pH 5.4	pH 7.4
0	0	0
1	2.75	12.76
3	6.36	21.5
6	12	32.5
12	20.4	45
24	28.5	53
48	33.25	62.5
72	39.2	70.8
96	43.5	77.5

##### ML framework

To mimic and predict pH-dependent release kinetics of oxaliplatin, supervised ML was employed using Python (v3.9) and the scikit-learn package (v1.4.0). The data set, representing release profiles at two pH conditions (10 time points per condition, 20 samples in total), was used entirely for training and interpolation in the model due to the limited sample size, which is routine practice to construct high-fidelity kinetic models from complete experimental data sampling. The primary aim was to characterize the intrinsic release mechanism and generate continuous predictions, rather than to forecast on unseen data. Hyperparameter tuning was conducted for all algorithms through systematic Grid Search with 5-fold cross-validation to prevent overfitting and obtain the optimal model configurations. Performance was assessed with the coefficient of determination (R²) on the training set, and the optimal hyperparameters were selected maximizing R² while model complexity was as low as possible. The trained models were then used to predict the release profiles at 500 equally spaced time points over the range 0–100 h, resulting in a high-resolution prediction of release kinetics. For mechanistic interpretation and understanding, SHAP analysis was performed using the shap Python package (v0.44.0) to determine the contribution of each feature (time, pH) to the model predictions and to identifying important transition points within the release profile.

#### Statistical analysis

All the experiments were performed in at least triplicate (*n* ≥ 3), and the data are presented as mean ± standard deviation (SD). GraphPad Prism software (version 8.0) was used for statistical analysis. In drug release study, cumulative release at 96 h at pH 5.4 and pH 7.4 was compared using an unpaired two-tailed Student’s t-test. Statistical difference between the treatment groups (free oxaliplatin and oxaliplatin-loaded nanomicelles) at different concentrations was determined in cytotoxicity assays (MTT) by two-way ANOVA followed by Sidak’s multiple comparisons test. p-value < 0.05 (*p* < 0.05) was considered statistically significant.

## Results and discussion

### Morphological analysis by SEM

**Fig. 1:**
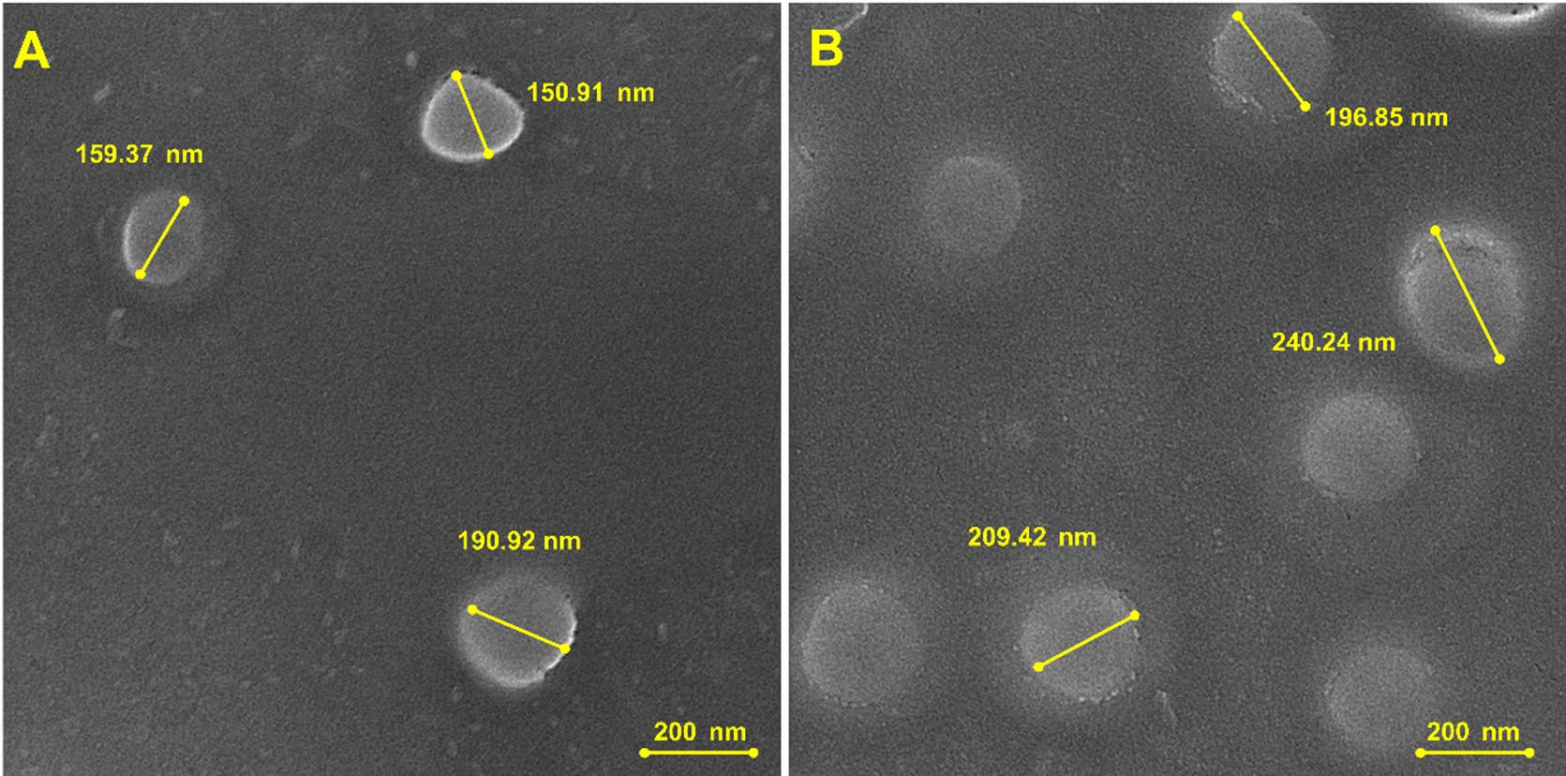
SEM images of (A) drug-free and (B) Oxaliplatin-loaded nanomicelles. The images confirm the spherical morphology and nanoscale size of the formulations. Scale bar: 200 nm.

SEM was used to determine the morphology and size of the prepared nanomicelles. The drug-free and Oxaliplatin-loaded nanomicelles, as shown in Fig. 1, exhibited spherical morphology and a smooth surface, characteristic of well-dispersed polymeric micelles. The SEM images also verify the lack of significant aggregation, which supports the good colloidal stability of the formulations. Particle sizes derived from SEM micrographs were in good agreement with DLS results. For the free drug nanomicelles, SEM indicated diameters of ca. 150–160 nm (Fig. 1A), in agreement with the Z-average diameter of 201 nm by DLS. Similarly, the diameters of Oxaliplatin-loaded nanomicelles by SEM ranged between 205 and 240 nm (Fig. 1B), consistent with the DLS measurement of 290 nm. The negligible difference in absolute size between SEM and DLS is to be expected and well documented; DLS provides the hydrodynamic diameter of particles in their solvated state, i.e., including the hydration shell and any surfactant corona, whereas SEM provides the size of dried core nanoparticle. The DLS and SEM data combined unequivocally demonstrate the successful synthesis of spherical, monodisperse, and nanoscale micellar carriers.

### Particle size evaluation

**Fig. 2:**
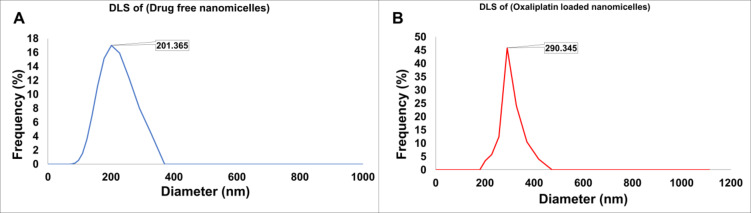
Particle size distribution of (A) drug-free and (B) Oxaliplatin-loaded nanomicelles as specified via Dynamic Light Scattering (DLS). The average hydrodynamic diameters were 201.365 nm and 290.345 nm, respectively.

The hydrodynamic diameter and size distribution of nanomicelles, both drug-free and Oxaliplatin-loaded, were determined by DLS analysis. Drug-free nanomicelles had a hydrodynamic diameter of 201.365 nm with a polydispersity index (PDI) of 0.067 (Fig. 2A), while Oxaliplatin-loaded nanomicelles exhibited an average diameter of 290.345 nm with a PDI of 0.023 (Fig. 2B). The low PDI values (PDI < 0.3) for both formulations confirm a monodisperse and uniform nanoparticle population, which is critical for consistent biodistribution and cellular uptake. The increase in particle size upon Oxaliplatin loading is likely due to the association of drug molecules with the micellar structure and potential swelling of the corona region.

### Drug encapsulation efficiency of oxaliplatin-loaded into nanomicelles

Oxaliplatin-loaded nanomicelles from the optimized aqueous hydration process were found to have the value of EE% as 40.2 ± 3.1% and DL% value as 3.5 ± 0.4%. The findings align with the anticipated integration efficiency of hydrophilic pharmaceuticals in Pluronic-based micellar systems, wherein drug loading predominantly transpires through connection with the hydrophilic corona rather than encapsulation within the hydrophobic core. The consistent yet mild EE% indicates the balance between drug-polymer affinity and the thermodynamic constraints of micellization. This optimal loading, through the rational aqueous-based route to preparation, facilitates the corona-association mechanism and provides a sufficient drug loading for therapeutic evaluation. The reproducible DL% of approximately 3.5% is a good drug content to carrier excipients balance, which is in favor of recommending prospective future pharmaceutical development with guaranteed nanocarrier stability and functional integrity.

### Drug release from oxaliplatin-incorporated nanomicelles

**Fig. 3:**
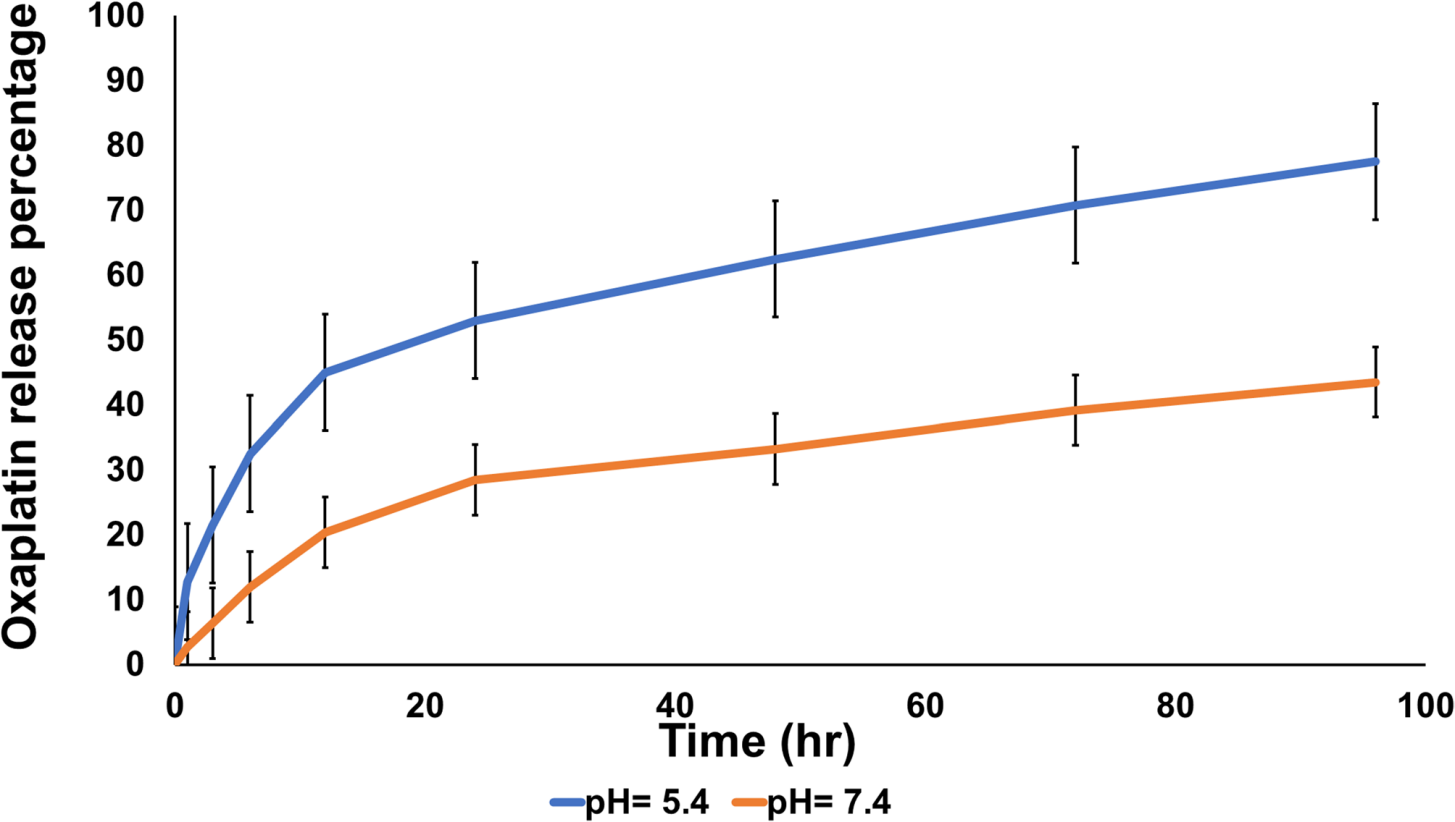
In vitro release profiles of Oxaliplatin from nanomicelles at pH 5.4 and pH 7.4. Data points represent mean ± standard deviation (*n* = 3).

Oxaliplatin release from nanomicelles had strong pH-dependent properties (Fig. 3). At physiological pH conditions (pH 7.4), release was slow and extended to the tune of 43.5% at 96 h. At acidic conditions mimicking the tumor microenvironment (pH 5.4), release was much more rapid to the value of 77.5% within the same period a 34% improvement. This profound enhancement, most pronounced in the initial 12 h whereby acidic conditions over-doubled the physiological rate, testifies further to the pH-responsiveness of the formula. Such heightened release at lower pH levels results from protonation-induced changes in the PEO corona that destabilize drug-polymer association. This differential release profile increases the potential of the system for targeted therapy by promoting preferential delivery of the drug to acidic tumor tissue and preventing premature release in normal physiological environments.

### Kinetic models of drug release

**Fig. 4:**
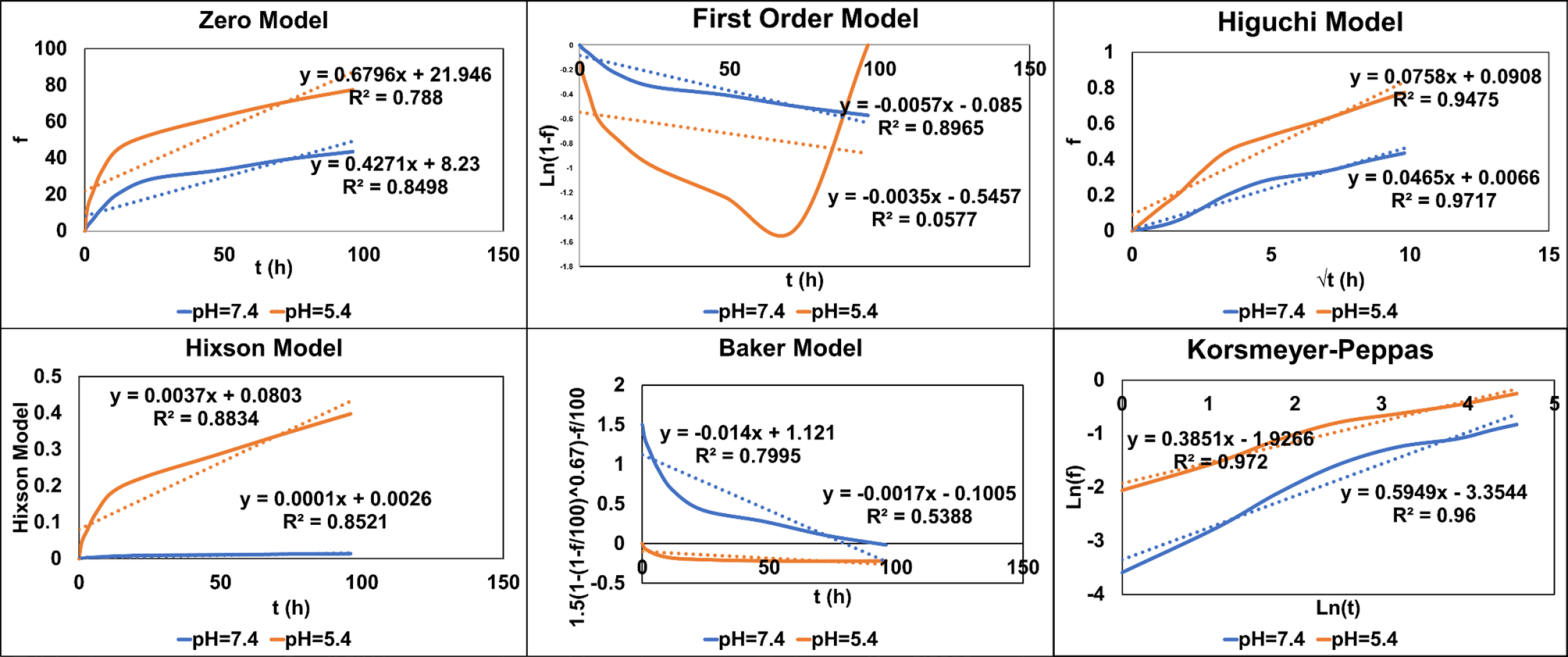
Kinetic modeling of oxaliplatin release from nanomicelles at pH 5.4 and pH 7.4 reveals diffusion as the predominant release mechanism, with variations in kinetics under different pH conditions.

The release kinetics of oxaliplatin from nanomicelles were analyzed using six mathematical models to elucidate the release mechanisms and pH-dependent behavior (Fig. 4; Table 2). The regression coefficients (R²) revealed distinct kinetic patterns under different pH conditions.

**Table 2:** Pharmacokinetic modeling of oxaliplatin release from nanomicelles.

Model	Equation	*R*² (pH 5.4)	*R*² (pH 7.4)
Zero-order	Ct = k_0_​t	0.8834	0.8498
First-order	ln (1- M_t_/M_∞_) = k_1_​t	0.8965	0.0577
Higuchi	Q = k_H_​t^0.5^	0.9475	0.9717
Hixson-Crowell	(1- M_t_/M_∞_)^1/3^ = k_H_​Ct	0.8521	0.8521
Baker-Lonsdale	Mt/M^∞^ = kBt^n^	0.7995	0.5388
Korsmeyer-Peppas	ln (M_t_/M_∞_) = nlnt + k	0.9720	0.9600

The release mechanisms were pH-dependent. The Higuchi model fitted extremely well at both pH levels (R² = 0.9475 at pH 5.4; R² = 0.9717 at pH 7.4), which suggested diffusion-controlled release as the prevailing mechanism. The Korsmeyer-Peppas model provided extra mechanistic information, and the release exponents were *n* = 0.3851 at pH 7.4 and *n* = 0.5949 at pH 5.4. In physiological pH (7.4), the value of n less than 0.45 suggests Fickian diffusion with drug release being concentration gradient-dominated. At acidic pH (5.4), the value of n between 0.45 and 0.89 suggests anomalous (non-Fickian) transport with incorporation of diffusion as well as relaxation processes of the polymer chain. The First-order model showed a poor correlation at pH 7.4 (R² = 0.0577) but good fit at pH 5.4 (R² = 0.8965) for the heterogeneity of release kinetics in acidic conditions. The Baker-Lonsdale model, which is relevant for spherical matrices, also better showed fit at pH 5.4 (R² = 0.7995) compared to pH 7.4 (R² = 0.5388), thus validating the influence of pH on release from the nanomicellar matrix. These results confirm that the release of oxaliplatin is via multiple kinetic pathways depending on the environment pH. The change from Fickian diffusion at physiological pH to anomalous transport in acidic pH supports the pH-sensitive characteristic of the nanomicelles, where drug release is enhanced in acidic environments through combined diffusion and erosion mechanisms and hence this kind of system is a suitable candidate for tumor-directed drug delivery.

### Machine-learning performance across pH conditions

To examine whether machine-learning (ML) methods can approximate oxaliplatin release kinetics, several regression models were benchmarked against classical kinetic fits. Performance metrics included the coefficient of determination (R²), root mean square error (RMSE), mean absolute error (MAE), and Akaike information criterion (AIC) (Tables 3 and 4). At both pH 5.4 and pH 7.4, ensemble models (Gradient Boosting, Random Forest) and support vector regression (SVR) produced very high in-sample R² values (> 0.97). For example, Gradient Boosting nearly reproduced the training data (R² ≈ 1.0, RMSE ≈ 0.0). However, these values should not be interpreted as evidence of predictive superiority. The dataset consisted of only nine experimental points per condition, making such models highly prone to overfitting. Perfect or near-perfect R² values mainly reflect memorization of the training set rather than genuine generalization capacity. By contrast, polynomial regression (R² ≈ 0.96–0.99) and the Korsmeyer–Peppas model (R² ≈ 0.89–0.96) yielded lower; but more realistic; fits, while retaining mechanistic interpretability. Importantly, classical models remain grounded in physicochemical processes (diffusion, relaxation, erosion), whereas ML models here serve primarily as flexible interpolators with limited mechanistic meaning. Thus, although ML models can interpolate observed profiles with high apparent accuracy, their predictive value beyond the measured timepoints is unproven without external validation. These results should therefore be regarded as exploratory and complementary to mechanistic kinetic modeling, not as replacements.

**Table 3:** Comparative model performance for oxaliplatin release at pH 5.4.

Model	*R*²	RMSE	MAE	AIC
Polynomial (deg3)	0.960	5.09	4.40	37.3
Random forest	0.973	4.15	3.78	425.6
Gradient boosting	1.000	0.00	0.00	383.4
SVR (RBF)	0.994	2.01	0.76	18.6
Korsmeyer–Peppas	0.960	–	–	–

**Table 4:** Comparative model performance for oxaliplatin release at pH 7.4.

Model	*R*²	RMSE	MAE	AIC
Polynomial (deg3)	0.990	1.55	1.23	15.9
Random Forest	0.976	2.38	1.98	415.6
Gradient Boosting	1.000	0.00	0.00	374.2
SVR (RBF)	1.000	0.10	0.10	−35.4
Korsmeyer–Peppas	0.892	–	–	–

#### Feature importance and SHAP analysis

To visualize model sensitivity, SHAP (Shapley Additive Explanations) analysis was performed. As expected, time and pH were the strongest contributors to variance. At pH 5.4, SHAP values peaked around 10–12 h and again near 72 h, coinciding with experimentally observed burst-to-sustained and sustained-to-plateau transitions. At pH 7.4, SHAP contributions were monotonic with time, reflecting the near-linear release regime. Nevertheless, these results should be regarded as descriptive visualizations rather than mechanistic evidence. With such a small dataset and nearly perfect in-sample fits, SHAP outputs may reflect mathematical inflection points in the fitted curves rather than true physicochemical transitions.

#### Quantitative mechanistic comparisons

When aligned with classical kinetic modeling, ML interpolations produced parameter values consistent with established mechanisms. At pH 7.4, a near-zero-order release constant was estimated:

4 $${Q}_{t}\:\:={k}_{H}\sqrt{t}$$

5 $$\frac{dQ}{dt}\:\:={k}_{0},\:\:\:\:\:\:\:\:{k}_{0} \approx\:0.42{\%}\cdot\:{eh}^{-1}$$

At pH 5.4, enhanced effective diffusivity during the mid-phase was consistent with anomalous (diffusion–relaxation) transport, as also captured by the Korsmeyer–Peppas model:

6 $$\:\frac{{M}_{t}}{{M}_{{\infty\:}}}=\text{k}{t}^{\text{n}}\:\:\:\:\:\:\:\left(n=0.48\:for\:diffusion,\:n=0.89\:for\:relaxation\right)$$

These values confirm that ML can reconstruct known kinetic patterns but do not imply independent mechanistic discovery.

#### Implications for formulation optimization

In principle, ML models could serve as surrogate interpolators for release profiles, enabling rapid in silico screening of formulation parameters (e.g., surfactant concentration, core composition, particle size). If validated on larger and more diverse datasets, such models could guide formulation adjustments to accelerate or delay transition phases depending on therapeutic needs. At present, however, this application remains speculative. Any predictive extrapolation beyond measured timepoints or mechanistic inference from SHAP should be interpreted with caution.

#### Limitations and recommended validation experiments

Key limitations include the very small dataset (nine points per pH condition), lack of external validation, and potential overfitting indicated by R² ≈ 1.0 values. While ensemble models reduced residual error, their apparent superiority is an artifact of interpolation, not generalization. Future studies should:

- Extend datasets across more pH values, particle compositions, and timepoints.
- Incorporate cross-validation or hold-out testing.
- Combine ML with physicochemical simulations to constrain predictions with mechanistic priors.

#### Implications for formulation optimization and predictive design

**Fig. 5:**
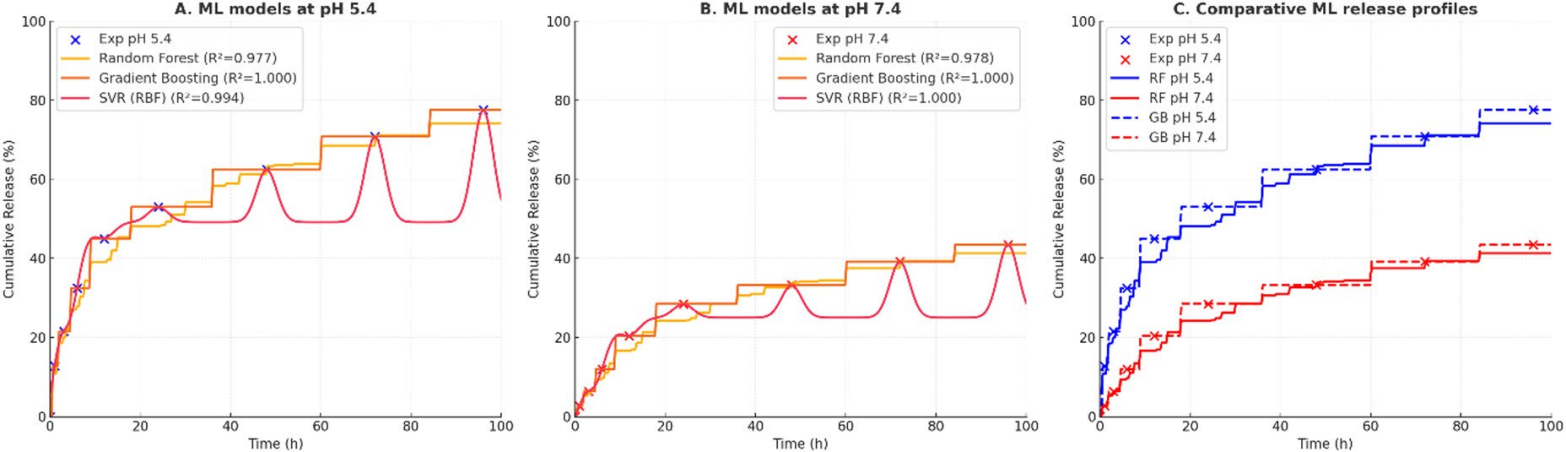
Model predictions of drug release at (**A**) pH = 5.4 and (**B**) pH = 7.4. (**C**) Comparative drug release profiles at different pH values.

The integrated interpretation of experimental and modeling results supports two distinct pH-dependent regimes (Fig. 5A–C). At pH 5.4 (acidic, tumor-like): Release follows a biphasic profile: an initial burst (0–12 h, ~ 30% release) consistent with Fickian diffusion, followed by a secondary phase (12–72 h, *n* ≈ 0.89) dominated by polymer relaxation and erosion, and plateau beyond 72 h (~ 77.5% at 96 h). ML interpolations aligned with these transitions but should be regarded as curve-fitting confirmations rather than mechanistic proof.

7 $$\frac{{M}_{t}}{{M}_{{\infty\:}}}=0.28{t}^{0.48}\left({R}^{2}=0.963\right)$$

8 $$\frac{{M}_{t}}{{M}_{{\infty\:}}}=0.14{t}^{0.89}\left({R}^{2}=0.991\right)$$

At pH 7.4 (physiological): Release is slower and near-linear (lag 0–6 h, ~ 13% release; 6–72 h, ~ 0.42%·h⁻¹; plateau ~ 43.5% at 96 h). This corresponds to diffusion-controlled kinetics consistent with Higuchi and Baker–Lonsdale models.

9 $$\:{Q}_{t}=0.51t+1.92$$

Thus, ML-assisted analysis reinforces but does not replace classical mechanistic modeling.

### Cytotoxicity of oxaliplatin-incorporated nanomicelles

**Fig. 6:**
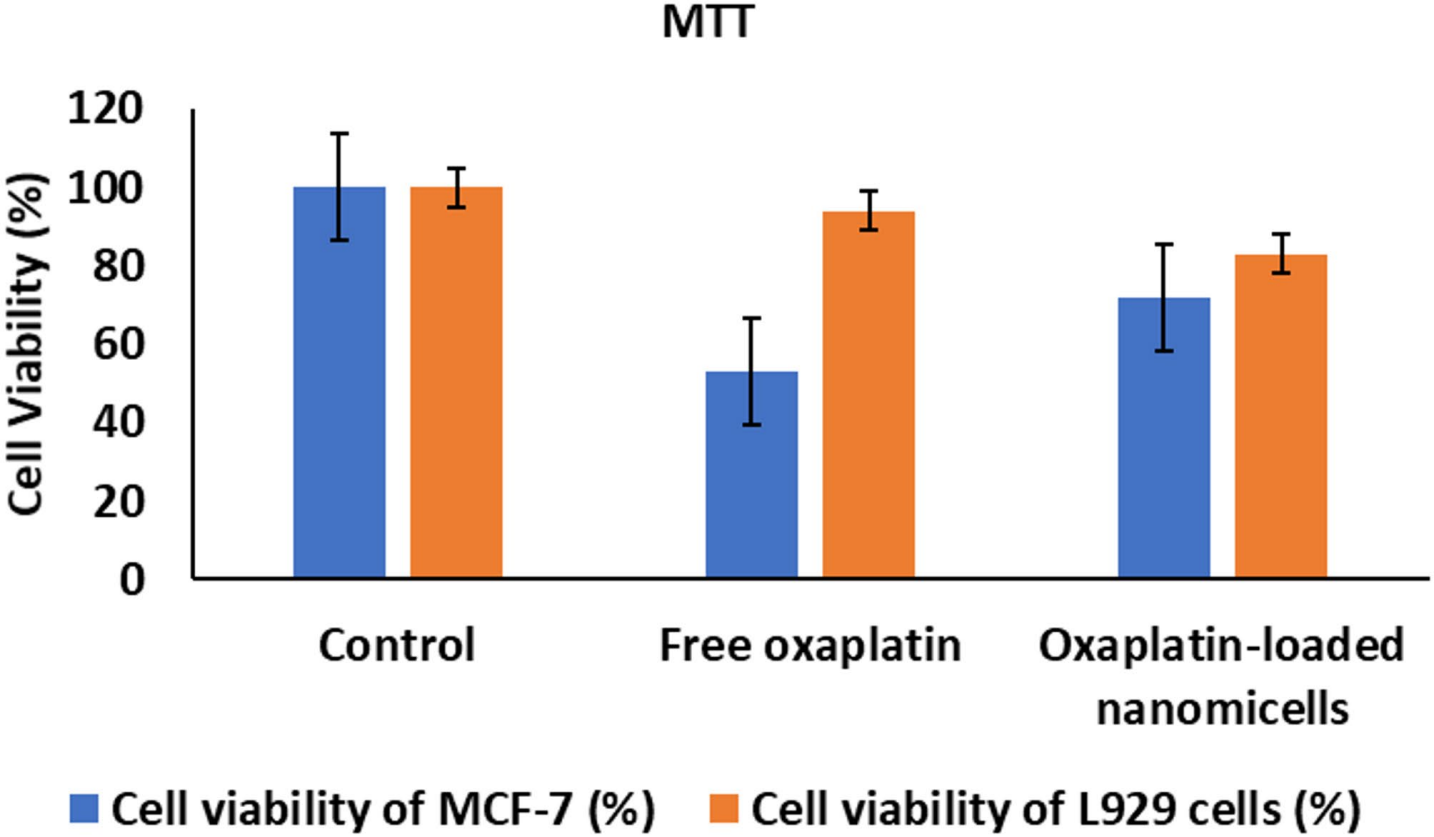
Cell viability of MCF-7 and L929 cells post-treatment with free Oxaliplatin and Oxaliplatin-loaded nanomicelles, assessed via MTT test. Data are expressed as mean ± standard deviation (*n* = 3).

The MTT test was put to use to determine the cytotoxicity of free Oxaliplatin and Oxaliplatin-loaded nanomicelles against MCF-7 breast cancer cells and L929 fibroblast cells. As can be seen in Fig. 6, Oxaliplatin nanomicelles exhibited greatly enhanced cytotoxic activity against MCF-7 breast cancer cells in comparison to free Oxaliplatin and concurrently also exhibited greatly reduced toxicity toward normal L929 fibroblasts. From this observation, it is clear that nanomicellar incorporation effectively regulated the drug’s biological activity, enhancing the drug’s efficacy against cancer cells while leaving the normal cells unharmed. The different response between the two cell types demonstrates a clear selective cytotoxicity, whereby the nanomicelles boost the therapeutic effectiveness against cancer cells while minimizing the undesirable off-target cytotoxicity against normal cells. These findings point out the potential of improving therapeutic efficacy with minimal adverse effects using Oxaliplatin-loaded nanomicelles.

Some studies highlight the potential of nanocarriers in improving the delivery and efficacy of oxaliplatin while reducing its side effects. However, they do not specifically focus on nanomicelles incorporated with oxaliplatin. K. Falahzadeh et al. study involves oxaliplatin-loaded chitosan nanoparticles embellished with cetuximab single-chain variable fragment (scFv) for targeting EGFR-overexpressing colorectal cancer cells. The nanoparticles showed increased cytotoxicity against HCT 116 cells^8^. Another study on self-assembled oxaliplatin nanomicelles (SANTA FE OXA) showed reduced cytotoxicity to normal cells while maintaining efficacy against cancer cells^9^. K. Wang et al. have utilized stearic acid-g-chitosan oligosaccharide (CSO-SA) micelles to encapsulate oxaliplatin, demonstrating enhanced cytotoxicity against cancer stem-like cells and colorectal cancer cells^10^.

Studies have shown that encapsulating oxaliplatin in PEGylated chitosan nanoparticles enhances its cytotoxic effect on MCF-7 cells. The combination of ascorbic acid and oxaliplatin in PEGylated nanoparticles enhances apoptotic effects compared to free drugs or non-PEGylated formulations. The IC50 values for oxaliplatin alone and incorporated in chitosan nanoparticles (CS NPs) were significantly higher than when incorporated in PEGylated chitosan nanoparticles (PEG-CS NPs), indicating improved efficacy with PEGylation^11^. Oxaliplatin tends to be more cytotoxic in MCF-7 cells than in triple-negative breast cancer (TNBC) cells like MDA-MB-231. This suggests that formulations like PEGylated chitosan nanoparticles might be particularly effective in enhancing oxaliplatin’s cytotoxicity in MCF-7 cells. Research on oxaliplatin combined with other drugs like olaparib and LY294002 demonstrates its cytotoxic effects on MCF-7 cells, though not specifically in nanomicelles^12^.

A comparison with other studies on the cytotoxicity of drug- incorporated nanomicelles in MCF-7 cells shows that, although not specifically focused on oxaliplatin formulations, other nanoparticle systems (e.g., doxorubicin-loaded nanoparticles) have exhibited variable cytotoxic effects in MCF-7 cells when contrasted with other cancer cell lines^13^. This investigation examined the cytotoxic effects of quercetin incorporated in solid lipid nanoparticles (QT-SLNs) on MCF-7 cells. The cytotoxicity of quercetin against MCF-7 cells is effectively increased by QT-SLNs, indicating the possibility of using them in cancer treatment^14^. According to other research, paclitaxel-loaded sterically stabilized phospholipid mixed nanomicelles dramatically boost the drug’s cytotoxicity in human breast cancer cell lines, including MCF-7 cells which are resistant and sensitive. Targeted delivery using VIP receptor-targeted nanomicelles further enhances cytotoxicity against multidrug-resistant (MDR) breast cancer cells^15^. This research evaluated the cytotoxicity of paclitaxel loaded onto dextran stearate micelles (Dex-SA/PTX) against MCF-7 cells. Dex-SA/PTX micelles exhibited more cytotoxicity than free PTX against MCF-7 breast cancer cells. The smaller size of these micelles enhanced their penetration into cells^16^. A meta-analysis assessed the cytotoxic effects of silver (Ag), zinc oxide (ZnO), and gold (Au) nanoparticles on MCF-7 cells. All three types of nanoparticles showed significant cytotoxicity at higher concentrations. Ag nanoparticles reduced cell viability more consistently, while Au and ZnO nanoparticles had variable effects^17^. In the study of Paclitaxel-Loaded Nanostructured Carrier Systems (NaCNs), PTX-loaded NaCNs exhibit upgraded cellular uptake, cytotoxicity, and in-vivo antitumor effects against free PTX. PTX-NaCNs’ IC50 values in both MDA-MB-231 and MCF-7 cell lines were noticeably lower than those of free PTX^18^.

In the other research investigates the cytotoxic effects of free and nanomicelle forms of curcuminoids on various cancer cell lines. While There was no discernible change in cytotoxicity among the two forms in most cell lines, nanomicelles showed lower toxicity in normal NIH3T3 cells^19^. Additionally, in cisplatin-resistant oral cancer cells, curcumin nanomicelles (CUR-NMs) demonstrated greater cytotoxicity and cellular absorption than plain curcumin. CUR-NMs increased the proportion of cells that underwent apoptosis^20^. In pH-Responsive Polymeric Nanomicelles for Paclitaxel Delivery investigations, F68-CAA-PTX nanomicelles demonstrated the highest cytotoxicity against MDA-MB-231 breast cancer cells, inducing significant apoptosis and ROS generation. These nanomicelles also showed enhanced effects in 3D tumor spheroids^21^. Table 5 comparing the cytotoxicity of nanomicelles incorporated with various drugs on MCF-7 and other cell lines:

**Table 5:** Competition the cytotoxicity of nanomicelles incorporated with drugs on cancer cell lines.

Drug incorporation	Cell line	IC50 value (µM)	Incorporated vs. free drug	Ref.
Oxaliplatin	HCT 116	Not specified, but incorporated shows-controlled release	incorporated has sustained release	^13^
Paclitaxel (PTX)	MCF-7	Lower IC50 for incorporated PTX	incorporated is more cytotoxic	^16^
Doxorubicin (DOX)	MCF7	1.2009 (free), 18.245 (incorporated)	Free DOX is more cytotoxic	^13^
Norcantharidin (NCTD)	HepG2	90.07 (incorporated), 117.50 (free)	incorporated is more cytotoxic	^22^
Norcantharidin (NCTD)	BEL-7402	63.20 (incorporated), 102.62 (free)	incorporated is more cytotoxic	^22^
Test compounds (S1-S4)	MCF-7	21.9–9.33 (incorporated), > 100 (free)	incorporated is more cytotoxic	^23^
Curcumin	Oral cancer cells	Higher cytotoxicity for incorporated curcumin	incorporated is more cytotoxic	^20^
Quercetin	Breast cancer cells	Lower IC50 for incorporated quercetin	incorporated is more cytotoxic	^24^

## Conclusion, limitation, and future directions

The study effectively developed surfactant-mediated nanomicelles as a selective and promising delivery system for Oxaliplatin for the treatment of breast cancer. The nanomicelles exhibited adequate physicochemical properties of nanoscale sizes, monodispersity, and significant pH-sensitive release behavior, which improves drug release under acidic, tumor-mimicking conditions. Such selective behavior was complemented by enhanced cytotoxicity against MCF-7 cancer cells and decreased off-target activity against normal fibroblasts. Furthermore, the use of machine learning models presented a robust complementary toolset, enabling high-fidelity release data interpolation and descriptive visualization of kinetic transitions via SHAP analysis. As the ML outputs are interpretative, rather than being mechanistically predictive due to dataset constraints, they underscore the vast potential for an integrated experimental-computational approach to simplify intricate drug delivery systems. While these are promising findings, the study does have some limitations that must be mentioned.

The use of a simple buffer system for release and cytotoxicity testing does not accurately reflect the complexity of the in vivo environment, particularly the potential impact of serum proteins and enzymes. The machine learning study was limited to two pH levels with nine time points each, which increased model overfitting and decreased generalizability. The study lacks in vivo pharmacokinetics, biodistribution, and anticancer activity validation, making it unable to report the critical micelle concentration (CMC), a key nanomicelle stability metric. Next studies must prove treatment efficacy and safety in serum-containing medium and in vivo models to overcome these limitations and build on these findings.

The machine learning model will require to be extended to more substantial and diverse datasets a series of polymer ratios, drug loads, and pHs combined with rigorous cross-validation to develop stable predictive models. On the development side, follow-up studies would involve the measurement of the CMC, the evaluation of long-term storage stability, and scalable process development for enabling clinical translation. Finally, ML combined with physiologically-based pharmacokinetic modeling or molecular dynamics simulation can improve mechanistic knowledge and rational nanomedicine design by predicting precisely.

## Data Availability

All data supporting the findings of this study are available within the paper.

## Declarations

AI tools were used for grammar and text enhancement, but the content was primarily authored by the writers.
